# Impacts of Climate Change on Public Health in India: Future Research Directions

**DOI:** 10.1289/ehp.1003000

**Published:** 2011-01-27

**Authors:** Kathleen F. Bush, George Luber, S. Rani Kotha, R.S. Dhaliwal, Vikas Kapil, Mercedes Pascual, Daniel G. Brown, Howard Frumkin, R.C. Dhiman, Jeremy Hess, Mark L. Wilson, Kalpana Balakrishnan, Joseph Eisenberg, Tanvir Kaur, Richard Rood, Stuart Batterman, Aley Joseph, Carina J. Gronlund, Arun Agrawal, Howard Hu

**Affiliations:** 1 Department of Environmental Health Sciences, School of Public Health, University of Michigan, Ann Arbor, Michigan, USA; 2 National Center for Environmental Health, Centers for Disease Control and Prevention, Atlanta, Georgia, USA; 3 University of Michigan Center for Global Health, Ann Arbor, Michigan, USA; 4 Indian Council of Medical Research, New Delhi, India; 5 Department of Evolutionary and Ecological Biology, University of Michigan, Ann Arbor, Michigan, USA; 6 Howard Hughes Medical Institute, Chevy Chase, Maryland, USA; 7 School of Natural Resources and Environment, University of Michigan, Ann Arbor, Michigan, USA; 8 University of Washington School of Public Health, Seattle, Washington, USA; 9 National Institute of Malaria Research, New Delhi, India; 10 Department of Epidemiology, School of Public Health, University of Michigan, Ann Arbor, Michigan, USA; 11 Department of Environmental Health and Engineering, Sri Ramachandra University, Chennai, India; 12 Department of Atmospheric, Oceanic and Space Sciences, University of Michigan, Ann Arbor, Michigan, USA

**Keywords:** air pollution, climate change, climate variability, health, heat, India, vector-borne, waterborne

## Abstract

**Background:**

Climate change and associated increases in climate variability will likely further exacerbate global health disparities. More research is needed, particularly in developing countries, to accurately predict the anticipated impacts and inform effective interventions.

**Objectives:**

Building on the information presented at the 2009 Joint Indo–U.S. Workshop on Climate Change and Health in Goa, India, we reviewed relevant literature and data, addressed gaps in knowledge, and identified priorities and strategies for future research in India.

**Discussion:**

The scope of the problem in India is enormous, based on the potential for climate change and variability to exacerbate endemic malaria, dengue, yellow fever, cholera, and chikungunya, as well as chronic diseases, particularly among the millions of people who already experience poor sanitation, pollution, malnutrition, and a shortage of drinking water. Ongoing efforts to study these risks were discussed but remain scant. A universal theme of the recommendations developed was the importance of improving the surveillance, monitoring, and integration of meteorological, environmental, geospatial, and health data while working in parallel to implement adaptation strategies.

**Conclusions:**

It will be critical for India to invest in improvements in information infrastructure that are innovative and that promote interdisciplinary collaborations while embarking on adaptation strategies. This will require unprecedented levels of collaboration across diverse institutions in India and abroad. The data can be used in research on the likely impacts of climate change on health that reflect India’s diverse climates and populations. Local human and technical capacities for risk communication and promoting adaptive behavior must also be enhanced.

## Climate Change and Human Health

Although low- and middle-income countries are responsible for only a small percentage of global greenhouse gas emissions, the adverse health effects associated with climate change will likely fall disproportionately on their populations. This inequity will further exacerbate global health disparities (McMichael et al. 2003; Patz and Olson 2006; Patz et al. 2007; Wiley and Gostin 2009). High-risk areas include those already experiencing a scarcity of resources, environmental degradation, high rates of infectious disease, weak infrastructure, and overpopulation (Patz et al. 2005; Wiley and Gostin 2009). In particular, tropical regions will experience significant changes in human–pathogen relationships because of climate change (Sattenspiel 2000). Changing temperatures and precipitation patterns linked to climate change will further affect health by changing the ecology of various vector-borne diseases, such as malaria, dengue, chikungunya, Japanese encephalitis, kala-azar, and filariasis (Bhattacharya et al. 2006; Dhiman et al. 2008). Vulnerable populations include the elderly, children, urban populations, and the poor (Ebi and Paulson 2010; O’Neill and Ebi 2009).

The goals of this report are to briefly summarize relevant literature and highlight the enormous challenges and opportunities for innovative research, with a particular focus on India. Such research is needed to pave the way for unique and pioneering solutions that can improve public health in the face of increasing climate variability. Therefore, we review the current state of the science relevant to the 2009 Joint Indo–U.S. Workshop on Climate Change and Health that was held in Goa, India, and then discuss the observed relationships between climate variability and human health, specifically in relation to the Indian subcontinent, highlighting future research directions.

Potential health impacts discussed at the Goa workshop fell into three categories: heat stress and air pollution, waterborne disease, and vector-borne disease focusing on malaria. Additional crosscutting sessions covered climate modeling and predictions for India, adaptation and vulnerability, surveillance and early warning systems, integration of spatial analysis, and bridging policy and science. We acknowledge that the potential physical and social impacts of climate change in India will likely be diverse, and that many additional important factors were not covered in our workshop, such as food yields, malnutrition, child growth, river flow, monsoon rain patterns, and freshwater availability. Nevertheless, we believe the Goa workshop served to target many of the major public health concerns associated with climate change and began the process of conceptualizing research needs and approaches that are integrative and achievable in low- and middle-income countries.

## Impacts in India

### The 2009 Joint Indo-U.S. Workshop on Climate Change and Health

The workshop was held in Goa, India, on 30 August through 2 September 2009; it was cosponsored by the University of Michigan’s Center for Global Health, the U.S. Centers for Disease Control and Prevention’s National Center for Environmental Health, and the Indian Council of Medical Research. Scientists from the cosponsoring institutions, along with other partners from academia, government, and nongovernmental organizations, met under the auspices of the existing Indo-U.S. Collaboration in Environmental and Occupational Health to discuss the current state of the science, identify gaps in understanding, and outline future research directions related to the human health effects of climate change in India. The focus was prediction and prevention in India, and discussions touched on the tremendous opportunities and significant challenges associated with designing, initiating, and conducting research, as well as pursuing related public health programming to improve public health infrastructure in the face of climate change.

### The scope of the problem and current research

#### Poverty and baseline vulnerability

**Figure 1:**
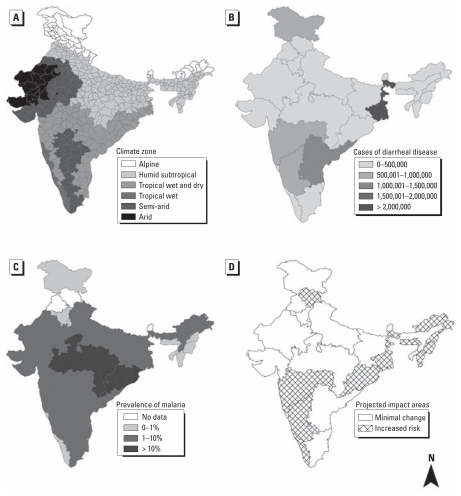
Interactions between climate and health in India. (*A*) Climate zones in India based on the Köppen classification that demonstrates the diversity of climates that exist in India [adapted from WikiProject India Maps and licensed under Creative Commons Attribution-Share Alike 3.0 (http://creativecommons.org/licenses/by-sa/3.0/)]. (*B*) State-specific estimates of cases of diarrheal disease across India in 2006 (adapted from Mandal 2008). (*C* and *D*) Regional estimates of malaria prevalence across India in 2002 (*C*) and regions in India where the prevalence of malaria is predicted to increase because of changes in climate (*D*) (adapted from Bhattacharya et al. 2006).

Many of the predicted effects of climate change are likely to become a reality in India. India is very diverse, geographically, climatically, and culturally (Figure 1A). It represents one-sixth of the world’s population, supported on 1/50 of the world’s land and 1/25 of the world’s water (Singh et al. 2010). With its huge and increasing population (~ 1.2 billion) and rate of urbanization, India is undergoing enormous change; climate change poses an overwhelming stressor that will magnify existing health threats. A greater understanding of the relationship between climate variability and human health in a country such as India could aid in the development of new prevention strategies and early warning systems, with implications throughout the developing world. Future studies must work to more explicitly define the relationship between climate variability and emerging and reemerging infectious diseases such as dengue, yellow fever, cholera, and the chikungunya virus (Shope 1991), as well as chronic diseases related to cardiovascular and respiratory illness, asthma, and diabetes. Millions of people below the poverty line and those in rural areas represent high-risk populations who are exposed to myriad health risks, including poor sanitation, pollution, malnutrition, and a constant shortage of clean drinking water. However, as awareness and public health infrastructure increase, the burden of climate-related disease may be negated (Dhiman et al. 2010).

#### Waterborne infectious disease

The burden of waterborne disease in India is enormous (Figure 1B). However, estimates vary widely because of a lack of reporting, poor surveillance, and minimal data infrastructure. A report from the Ministry of Health and Family Welfare estimates that nearly 40 million people are affected by waterborne disease every year that places a large burden on both the health sector and the economic sector. As a consequence, approximately 73 million workdays or US$600 million are lost each year (Mandal 2008). Although the World Health Organization (WHO) estimates that 900,000 Indians die each year from drinking contaminated water and breathing polluted air (WHO and UNICEF 2000), the Indian Ministry of Health estimates 1.5 million deaths annually among 0- to 5-year-old children. Cholera provides another example, with approximately 5 million cases reported by WHO each year; however, this estimate is thought to be a gross underestimation of the true burden of cholera because of a lack of surveillance and underreporting on the Indian subcontinent (Zuckerman et al. 2007). Approximately 73% of the rural population in India does not have proper water disinfection, and 74% do not have sanitary toilets (International Institute for Population Sciences and Macro International 2007). Freshwater availability in India is also a concern; available water is expected to decrease from 1,820 m^3^ per capita to < 1,000 m^3^ by 2025 in response to the combined effects of population growth and climate change (Intergovernmental Panel on Climate Change 2007).

Research in this area must be both temporally and spatially specific. Furthermore, it requires local monitoring of the appropriate climate and disease variables (Patz et al. 2002) because underreporting impedes the development of effective prevention strategies. It is critical to build a data infrastructure and conduct such research in India so that region-specific models based on climate and health can be developed. A systems approach focusing on health outcomes is critical to the success of future research in this area (Batterman et al. 2009). As prediction models evolve, region-specific action plans and adaptation strategies can be developed.

#### Heat stress and air pollution

The summer of 2010 was the hottest summer on record in India, with temperatures approaching 50°C (122°F); the effects were far-reaching, including hospitalization because of heatstroke, suffering of livestock, and severe drought in some regions that affected health as well as agriculture (Burke 2010). Research linking temperature and health effects in India is sparse. However, in a study of 12 international urban areas that included Delhi, McMichael et al. (2008) found a 3.94% [95% confidence interval (CI), 2.80–5.08%] increase in mortality for each 1°C increase above 29°C. Hajat et al. (2005) reported that individuals in the 0- to 14-year-old age group had greater vulnerability to temperature increases in Delhi than did those in the 15- to 64-year-old age group or in the ≥ 65-year-old age group. These findings are in direct contrast with results from cities in Europe and the United States that consistently identify the elderly as the more vulnerable age group. Hajat et al. (2005) also found that harvesting (whereby increases in mortality on one day are followed by substantial decreases in mortality in subsequent days) accounted for almost all temperature-related mortality in London, whereas in Delhi, the increase in mortality due to high temperatures was not followed by an immediate drop in mortality. This suggests that in Delhi, individuals who died on days with higher temperatures were not already near death.

Limited work has been conducted on the combined effects of weather, climate variability, and increased air pollution in India (Agarwal et al. 2006; Karar et al. 2006). One study that investigated the effects of air pollution on respiratory disease found that emergency department visits increased by approximately 20% because of high levels of pollutants in Delhi (Pande et al. 2002). In a second study based in Chennai, India, Ghosh et al. (2010) concluded that short-term exposure to particulate matter ≤ 10 μm in aerodynamic diameter (PM_10_) resulted in an estimated risk ratio of 1.0044 (95% CI, 1.002–1.007) per a 10 μg/m^3^ increase in daily average concentrations; this risk estimate is comparable to similar estimates from other countries. An important contribution of this study, relevant to other low- and middle-income countries, was the development of new methods to address specific limitations of routinely collected data such as missing measurements and small footprints of air pollution monitors, but the link to temperature remains to be explored. Some work has been done on seasonal air quality monitoring (Pulikesi et al. 2006); however, the relationship of temperature, ozone, and health requires further investigation (Doherty et al. 2009). Indoor air pollution presents yet another major health threat, with 32% of deaths in South Asia attributable to the burning of solid fuels in poor, small, unventilated houses (Smith 2000; WHO 2004). Whether these health risks will be exacerbated as a result of climate change is yet to be determined, but cobenefit interventions aimed at reducing the health impacts associated with indoor air pollution, decreasing the release of green house gases from the burning of solid fuel, and preventing deforestation by introducing alternative, more efficient stoves and fuels will have serious implications for health and society.

#### Vector-borne disease

India has approximately 2 million confirmed cases of malaria per year (Kumar et al. 2007). Like most infectious diseases, prevalence varies by region (Figure 1C,D). Although WHO concludes that approximately 15,000 individuals die from malaria each year in India (WHO 2008), a recent study by Dhingra et al. (2010) estimates approximately 200,000 malaria deaths per year in India before 70 years of age and 55,000 in early childhood. As Dhingra et al. (2010) suggest, accurate estimation of malaria mortality in India is difficult because correctly diagnosed episodes are successfully treated and do not result in death; in fatal cases without medical intervention, malaria is easily mistaken for some other life-threatening fever; and in most rural areas where death from malaria is common, proper medical attention at the time of death is uncommon. These challenges, which hold true in many developing countries, make it difficult to use hospital-based data to assess the association between climate variability and malaria, because disease burden may be vastly underestimated.

In India, 65% of malaria cases are reported from six regions (Orissa, Jharkhand, Madya Pradesh, Chattsgarh, West Bengal, and the North East). In Orissa, the disease has much more serious proportions than even in sub-Saharan Africa (Narain 2008). A 2001 WHO report estimated the disability-adjusted life years lost because of all vector-borne diseases in the country to be 4.2 million, and malaria is believed to account for nearly half of this (Dash et al. 2008). The emergence and rapid spread of drug-resistant strains of malaria further compound the problem. Chloroquine used to be the drug of choice for all kinds of malaria and was highly prescribed in India until 1973, when resistance was detected in *Plasmodium falciparum*. Chloroquine is no longer as effective, with increasing reports of *Plasmodium vivax* developing resistance (Dash et al. 2008). In addition, the use of chloroquine, which selects against *P. vivax*, has allowed *P. falciparum* to become the dominant parasite (Singh et al. 2004), a pattern with important epidemiological consequences, because it is the most virulent form of malaria in the region.

In arid and semiarid regions of India, where malaria is epidemic, rainfall variability has been shown to drive the interannual variability of the disease (Akhtar and McMichael 1996; Bouma and van der Kaay 1994; Laneri et al. 2010) and was the basis of one of the first early-warning systems for the disease in this region. Evidence suggests that rainfall variability plays an important role and that a long-term trend in increasing temperature during the 20th century is sufficient to significantly increase the abundance of vectors (Pascual et al. 2009). Monthly parasite incidence was positively correlated with temperature, precipitation, and humidity (Devi and Jauhari 2006). The implications of this association as it relates to long-term climate change remain an important open question. For other regions of India, monsoonal rains have shown an increase in the frequency and magnitude of extreme rain events, whereas the frequency of moderate events has been decreasing, with no significant change in the mean in the last 50 years (Goswami et al. 2006). Temperature plays a major role, especially at high altitudes, preventing epidemic malaria from spreading into the highest altitude regions. The consequences of climate change in highland regions is an important open question based on future temperature predictions in these regions (Beig G, unpublished data). Little is known about the influence of climate variability or climate change on the prevalence of malaria in Indian urban areas (Kumar et al. 2007). The issue of urban malaria becomes even more important when considering the rapid expansion of urban and periurban environments, water storage techniques, and rising poverty levels.

### The need for adaptation

Although adaptation to climate impacts has attracted substantial attention recently, the effectiveness of specific strategies in relation to greater resilience of public health systems remains underinvestigated. Adapting to climate change will be necessary and will occur at physiological, behavioral, social, institutional, and organizational scales. To take advantage of already ongoing adaptations for creating more effective public health responses to climate change impacts—especially for poor rural communities whose access to health care is extremely limited even in the current policy environment—developing a baseline understanding of the region-specific demographic, social, and ecological determinants of health will be necessary. In designing public health responses, factors that must be considered include the population’s age structure, socioeconomic profile, baseline prevalence of climate-sensitive diseases, public awareness of risk, the built environment, existing infrastructure, available public health services, and autonomous responses to climate impacts on health that households and communities might undertake by themselves (McMichael 2004). Furthermore, adaptation strategies in response to climate variability and change must be designed on specific temporal and spatial scales relevant to India. Taking steps now to adjust to current climate variability and modifying existing programs to address the anticipated impacts of climate change will make future adaptation strategies more effective (Ebi et al. 2006). The same changes may also aid in reaching additional environmental and social objectives, such as more equitable education, empowerment of women, and improved sanitation. These community-based initiatives should be complemented by government interventions. A variety of stakeholders, including those who will be affected most by climate change impacts, must be involved in the problem-solving process to enhance human and technical capacity across sectors at both local and national levels (Agrawal 2009; Ebi and Semenza 2008). Failure to invest now will likely increase the severity of consequences in the future (Haines et al. 2006).

Potential adaptation strategies in India could focus on controlling infectious diseases by removing vector breeding sites, reducing vector–human contact via improved housing, and coordinating monitoring of mosquitoes, pathogens, and disease burden. Another potential focus area for adaptation could be improving sanitation and drinking water by supporting inexpensive and effective water treatment and increasing rainwater harvesting, safe storage, and gray-water reuse. In some areas, the focus may shift to flood, heat wave, and emergency preparedness, including strategies to address the additional risks placed on displaced populations from these and other climate-sensitive hazards. One possible outcome could be the development of an integrated early warning system, emergency response plans, and refugee management plans, along with increased capacity to provide shelter, drinking water, sanitation, and sustainable agricultural products to the most vulnerable populations.

### Current surveillance and data sources

Successful work in this area will require the health community to partner closely with climate scientists and development professionals to move beyond the assessment of climate variability and disease outcomes to predictive models accounting for climate change to facilitate targeted adaptation. Partnerships with both the government and nongovernment sector will also be necessary. An integrated disease surveillance system already exists under the director general of health services; any new work on climate change and health should be linked to the already existing system. The Energy and Resources Institute (TERI) in Delhi, India, is one example of such a group linking research and action by increasing awareness within India and sharing the “developing country” perspective on climate change with the rest of the world. Activities at TERI range from operating as a think tank at the local level to forging global alliances for collaborative research. Collaborative work is also being conducted at the National Institute of Malaria Research in partnership with Mercedes Pascual at the University of Michigan to assess the impacts of climate change on malaria and dengue at a national scale and to develop adaptation strategies. In addition, this same partnership is developing an evidence-based assessment of biophysical determinants affecting malaria in the northeastern states of India and a framework for malaria control under changing climate scenarios. Several other nongovernmental organizations are working on climate change in India, such as the Local Governments for Sustainability with a regional office in New Delhi; Resources for the Future, which is partnering closely with the Public Health Foundation of India; and Toxics Link, which is working on traditional environmental health with a new focus on climate change.

Retrospective studies investigating climate variability and health dominate the literature, leaving predictive and prospective studies related to climate change open to be explored. However, both prospective and retrospective studies need high-quality data. Working groups at the Goa workshop were able to identify existing and relevant long-term data sets that can be used for environmental epidemiological analysis. For example, both the Indian Institute of Tropical Meteorology (IITM 2010) and the India Meteorological Department (2010) have useful meteorological data with varying degrees of access. Additional government surveys such as the Census of India and the National Family Health Survey, India provide important information on social and economic variables. In some cases, individual investigators have accessed government hospital data sets and have daily all-cause mortality, albeit over a limited geography. The same goes for air pollution data, such as data on particulate matter (PM), which have been accessed at certain locations of interest, such as Chennai (Ghosh et al. 2010). The use of exposure and emission models can help to fill in where air pollution data are missing; however, consistent monitoring of PM, ozone, and nitrogen oxides over a greater geographic area is needed. In cases where the data already exist, more work is needed to identify and access this type of long-term data, creating uniform repositories. In cases where it does not exist, surveillance and monitoring of relevant variables will be critical to the success of future prospective climate and health research endeavors. Furthermore, regional climate models for India such as PRECIS (Providing Regional Climates for Impacts Studies) developed at the IITM must be integrated with health data if we are going to transition away from surveying the health effects associated with climate variability to predicting the effects of climate change.

Changes to the current information infrastructure needed for this effort will depend on new or enhanced collaborations across multiple disciplines and among diverse institutions. Given the region-specific nature of the relationship between climate variability and health, further research is required throughout India. Satellite and geospatial technology may provide new insights regarding the geographic distribution of risk and disease. Integration of social, demographic, and land cover data with health data will aid in describing a holistic health scenario, which will help identify sustainable health solutions. These research needs and methodological limitations are relevant to many low- and middle-income countries. India, with its current health infrastructure and large population, can serve as an important natural laboratory for developing relevant strategies for promoting and managing climate health research in the developing world.

## Recommendations

As a result of the Goa workshop and subsequent discussions, the following recommendations to advance research relevant to climate change and human health were proposed.

### Environmental monitoring and surveillance

There is a great need to improve environmental monitoring and surveillance systems in low- and middle-income countries such as India. New research initiatives should focus on collecting high-quality, long-term data on climate-related health outcomes with the dual purpose of understanding current climate–health associations and predicting future scenarios. Health outcomes of interest, for which such data should be collected, include total morbidity and mortality and noncommunicable diseases such as cardiovascular, respiratory, and circulatory diseases and asthma, as well as infectious diseases such as cholera, malaria, tuberculosis, typhoid, hepatitis, dysentery, tick-borne encephalitis, and other vector-borne and waterborne diseases. Such monitoring also requires the collection of appropriate climatic (e.g., temperature and precipitation) and nonclimatic data (e.g., ozone). Surveillance of extreme weather conditions and risk indicators such as mosquito abundance or pathogen load is also necessary. Such data gathering should occur in conjunction with already existing public health programs and health centers. Where the necessary public health infrastructure does not exist, the anticipated risks associated with climate change should motivate international action to build such infrastructure. The collection of such diverse data necessitates the creation of linkable and documented repositories for meteorological, air pollution, and health data. Such a virtual network, or clearinghouse, will help researchers as well as practitioners as they work toward defining climate–health associations and designing effective interventions. Such monitoring provides the information and feedback necessary to take action in response to the anticipated changes in climate and burden on the public health infrastructure.

### Geospatial technology

Geographic information systems and spatial analysis must be further developed; they are very useful tools when conducting vulnerability assessments, assessing environmental exposures, prioritizing research, and disseminating findings to decision makers and the public alike (Jerrett et al. 2010). Remote sensing and environmental monitoring are particularly useful to catalog variables such as air pollution and heat exposure. Social data from census and surveys, which can be layered with the exposure data using geographic information systems, provide information on sensitivity and adaptive capacity, at both individual and community levels. Data on land use and land cover can provide additional information on relevant environmental factors that influence risk and vulnerability.

Such a spatial information infrastructure provides the necessary data-integration framework to combine information on human–environment interactions from a variety of sources. Vulnerability assessments can be conducted spatially and temporally through integration of such social and environmental data. Risk maps can incorporate social and ecological risk factors in an attempt to characterize the existing spatial heterogeneity. This is a very effective tool when predicting prevalence, targeting resource distribution, and designing control programs for different infectious diseases such as malaria (Ageep et al. 2009; Haque et al. 2010; Reid et al. 2010; Tonnang et al. 2010). An example of such work, which grew out of the Goa workshop, will focus on the effect of socioeconomic status on the association between climate and malaria.

### Human and technical capacity

For these new surveillance methods and analytical techniques to be effective, countries like India will need to enhance their human and technical capacity for risk communication. This could take the form of public education on climate change and associated health impacts to enhance awareness and to influence lifestyle, behavior, and individual choices to protect and improve health. Such health promotion materials could manifest as low-tech flyers and advertisements as well as more high-tech materials including web-based and mobile-phone–based alerts. On the other end of the spectrum, developing capacity could take on a more holistic approach, such as region- and city-specific climate action plans and early warning system for heat stress events, droughts, hurricanes, and floods.

## Conclusions

Studies of climate variability and human health indicate a great deal of heterogeneity in the reported associations. This heterogeneity is partially due to differences in study design, but climatic and socioeconomic differences that vary by location also influence the burden of disease. It is not clear whether results from one region can be extrapolated to others. Therefore, it is important to develop a comprehensive catalog of climate change and associated health outcomes across the range of environments and populations likely to be affected. A better understanding of the effects of climate change on health in India will be best achieved through studies specific to climates and populations in India.

In 2008 India developed the National Action Plan on Climate Change, promising further enhancement of ecological sustainability as part of India’s development path, signaling their involvement in the international discussion on climate change. Countries like India have a tremendous opportunity to guide our future trajectory regarding sustainable development and adaptation to climate change, but it will take the combined effort of policy makers and scientists from around the world to address the complex challenges associated with climate change and human health.

In conclusion, innovative, multidisciplinary investigations using environmental epidemiologic methods to elucidate health risks posed by climate variability—and subsequent climate change—in regions such as India are possible. However, such work will require expanded partnerships among researchers, governments, and communities to develop a cobenefit strategy that addresses public health challenges and risks associated with climate change. Adoption and implementation of these research initiatives will provide the necessary tools and infrastructure to pose interesting scientific questions and design effective solutions to the complex issues imposed by climate change.

## References

[b1-ehp-119-765] Agarwal R, Jayaraman G, Anand S, Marimuthu P (2006). Assessing respiratory morbidity through pollution status and meteorological conditions for Delhi. Environ Monit Assess.

[b2-ehp-119-765] Ageep TB, Cox J, Hassan MM, Knols BGJ, Benedict MQ, Malcolm CA (2009). Spatial and temporal distribution of the malaria mosquito *Anopheles arabiensis* in northern Sudan: influence of environmental factors and implications for vector control. Malar J.

[b3-ehp-119-765] Agrawal A, Mearns R, Norton A (2009). Local institutions and adaptation to climate change. Social Dimensions of Climate Change: Equity and Vulnerability in a Warming World.

[b4-ehp-119-765] Akhtar R, McMichael AJ (1996). Rainfall and malaria outbreaks in western Rajasthan. Lancet.

[b5-ehp-119-765] Batterman S, Eisenberg J, Hardin R, Kruk ME, Lemos MC, Michalak AM (2009). Sustainable control of water-related infectious diseases: a review and proposal for interdisciplinary health-based systems research. Environ Health Perspect.

[b6-ehp-119-765] Bhattacharya S, Sharma C, Dhiman RC, Mitra AP (2006). Climate change and malaria in India. Curr Sci.

[b7-ehp-119-765] Bouma MJ, van der Kaay HJ (1994). Epidemic malaria in India and the El Nino Southern Oscillation. Lancet.

[b8-ehp-119-765] Burke J (2010). Hundreds Die in Indian Heatwave. Guardian 30 May.

[b9-ehp-119-765] Dash AP, Valecha N, Anvikar AR, Kumar A (2008). Malaria in India: challenges and opportunities. J Biosci.

[b10-ehp-119-765] Devi NP, Jauhari RK (2006). Climatic variables and malaria incidence in Dehradun, Uttaranchal, India. J Vector Borne Dis.

[b11-ehp-119-765] Dhiman RC, Pahwa S, Dash AP (2008). Climate change and Malaria in India: interplay between temperature and mosquitoes. Regional Health Forum.

[b12-ehp-119-765] Dhiman RC, Pahwa S, Dhillon GPS, Dash AP (2010). Climate change and threat of vector-borne disease in India: are we prepared?. Parasitol Res.

[b13-ehp-119-765] Dhingra N, Jha P, Sharma VP, Cohen AA, Jotkar RM, Rodriguez PS (2010). Adult and child malaria mortality in India: a nationally representative mortality survey. Lancet.

[b14-ehp-119-765] Doherty RM, Heal MR, Wilkinson P, Pattenden S, Vieno M, Armstrong B (2009). Current and future climate- and air pollution-mediated impacts on human health. Environ Health (suppl 1).

[b15-ehp-119-765] Ebi KL, Kovats RS, Menne B (2006). An approach for assessing human health vulnerability and public health interventions to adapt to climate change. Environ Health Perspect.

[b16-ehp-119-765] Ebi KL, Paulson JA (2010). Climate change and child health in the United States. Curr Probl Pediatr Adolesc Health Care.

[b17-ehp-119-765] Ebi KL, Semenza JC (2008). Community-based adaptation to the health impacts of climate change. Am J Prev Med.

[b18-ehp-119-765] Ghosh S, Johnson P, Ravinder S, Chakraborty M, Mittal M, Balakrishnan K (2010). Development and application of spatially disaggregated exposure series in time-series analyses of air pollution-related health effects in Chennai, India. Epidemiology.

[b19-ehp-119-765] Goswami BN, Venugopal V, Sengupta D, Madhusoodanan MS, Xavier PK (2006). Increasing trend of extreme rain events over India in a warming environment. Science.

[b20-ehp-119-765] Haines A, Kovats RS, Campbell-Lendrum D, Corvalan C (2006). Climate change and human health: impacts, vulnerability, and public health. Public Health.

[b21-ehp-119-765] Hajat S, Armstrong BG, Gouvia N, Wilkinson P (2005). Mortality displacement of heat-related deaths: a comparison of Delhi, São Paulo, and London. Epidemiology.

[b22-ehp-119-765] Haque U, Magalhães RJS, Reid HL, Clements ACA, Ahmed SM, Islam A (2010). Spatial prediction of malaria prevalence in an endemic area of Bangladesh. Malar J.

[b23-ehp-119-765] India Meteorological Department (2010). India Meteorological Department, Pune.

[b24-ehp-119-765] Indian Institute of Tropical Meteorology (2010). IITM: A World Centre of Excellence in Basic Research on the Ocean–Atmosphere Climate System Required for Improvement of Weather and Climate Forecasts.

[b25-ehp-119-765] Pachauri RK, Reisinger A, Intergovernmental Panel on Climate Change, Core Writing Team (2007). Climate Change 2007: Synthesis Report. Contribution of Working Groups I, II and III to the Fourth Assessment Report of the Intergovernmental Panel on Climate Change.

[b26-ehp-119-765] International Institute for Population Sciences and Macro International (2007). National Family Health Survey (NFHS-3), 2005–06: India.

[b27-ehp-119-765] Jerrett M, Gale S, Kontgis C (2010). Spatial modeling in environmental and public health. Int J Environ Res Public Health.

[b28-ehp-119-765] Karar K, Gupta AK, Kumar A, Biswas AK (2006). Seasonal variations of PM_10_ and TSP in residential and industrial sites in an urban area of Kolkata, India. Environ Monit Assess.

[b29-ehp-119-765] Kumar A, Valecha N, Jain T, Dash AP (2007). Burden of malaria in India: retrospective and prospective view. Am J Trop Med Hyg.

[b30-ehp-119-765] Laneri K, Bhadra A, Ionides EL, Bouma M, Dhiman RC, Yadav RS (2010). Forcing versus feedback: epidemic malaria and monsoon rains in northwest India. PLoS Comput Biol.

[b31-ehp-119-765] Mandal K (2008). Drinking Water Supply vis-a-vis Technological Interventions for Social Empowerment of Rural India.

[b32-ehp-119-765] McMichael A, Ezzati M, Lopez A, Rodgers A, Murray C (2004). Climate change. Comparative Quantification of Health Risks: Global and Regional Burden of Disease due to Selected Major Risk Factors.

[b33-ehp-119-765] McMichael AJ, Campbell-Lendrum DH, Corvalán CF, Ebi KL, Githeko A, Scheraga JD (2003). Climate Change and Human Health—Risks and Responses.

[b34-ehp-119-765] McMichael AJ, Wilkinson P, Kovats RS, Pattenden S, Hajat S, Armstrong B (2008). International study of temperature, heat and urban mortality: the “ISOTHURM” project. Int J Epidemiol.

[b35-ehp-119-765] Narain JP (2008). Malaria in the South-East Asia region: myth and the reality. Indian J Med Res.

[b36-ehp-119-765] O’Neill MS, Ebi KL (2009). Temperature extremes and health: impacts of climate variability and change in the United States. J Occup Environ Med.

[b37-ehp-119-765] Pande JN, Bhatta N, Biswas D, Pandey RM, Ahluwalia G, Siddaramaiah NH (2002). Outdoor air pollution and emergency room visits at a hospital in Delhi. Indian J Chest Dis Allied Sci.

[b38-ehp-119-765] Pascual M, Dobson AP, Bouma MJ (2009). Underestimating malaria risk under variable temperatures. Proc Natl Acad Sci USA.

[b39-ehp-119-765] Patz JA, Campbell-Lendrum D, Holloway T, Foley JA (2005). Impact of regional climate change on human health. Nature.

[b40-ehp-119-765] Patz JA, Gibbs HK, Foley JA, Rogers JV, Smith KR (2007). Climate change and global health: quantifying a growing ethical crisis. EcoHealth.

[b41-ehp-119-765] Patz JA, Hulme M, Rosenzweig C, Mitchell TD, Goldberg RA, Githeko AK (2002). Climate change: regional warming and malaria resurgence. Nature.

[b42-ehp-119-765] Patz JA, Olson SH (2006). Climate change and health: global to local influences on disease risk. Ann Trop Med Parasitol.

[b43-ehp-119-765] Pulikesi M, Baskaralingam P, Elango D, Rayudu VN, Ramamurthi V, Sivanesan S (2006). Air quality monitoring in Chennai, India, in the summer of 2005. J Hazard Mater.

[b44-ehp-119-765] Reid H, Haque U, Clements ACA, Tatem AJ, Vallely A, Ahmed SM (2010). Mapping malaria risk in Bangladesh using Bayesian geostatistical models. Am J Trop Med Hyg.

[b45-ehp-119-765] Sattenspiel L (2000). Tropical environments, human activities, and the transmission of infectious diseases. Am J Phys Anthropol.

[b46-ehp-119-765] Shope R (1991). Global climate change and infectious diseases. Environ Health Perspect.

[b47-ehp-119-765] Singh MR, Upadhyay V, Mittal AK (2010). Addressing sustainability in benchmarking framework for Indian urban water utilities. J Infrastr Systems.

[b48-ehp-119-765] Singh N, Chand SK, Mishra AK, Nagpal AC (2004). Migration malaria associated with forest economy in central India. Curr Sci.

[b49-ehp-119-765] Smith KR (2000). National burden of disease in India from indoor air pollution. Proc Natl Acad Sci USA.

[b50-ehp-119-765] Tonnang HEZ, Kangalawe RYM, Yanda PZ (2010). Predicting and mapping malaria under climate change scenarios: the potential redistribution of malaria vectors in Africa. Malar J.

[b51-ehp-119-765] WHO (World Health Organization) (2004). World Health Report 200—Changing History.

[b52-ehp-119-765] WHO. (World Health Organization) (2008). World Malaria Report 2008.

[b53-ehp-119-765] WHO and UNICEF (World Health Organization and United Nations Children’s Fund) (2000). Water Sanitation and Health (WSH). Global Water Supply and Sanitation Assessment 2000 Report.

[b54-ehp-119-765] Wiley LF, Gostin LO (2009). The international response to climate change: an agenda for global health. JAMA.

[b55-ehp-119-765] Zuckerman JN, Rombo L, Fisch A (2007). The true burden and risk of cholera: implications for prevention and control. Lancet Infect Dis.

